# 1114. Comparative analysis between Polymerized Type I Collagen and Baricitinib as potential treatment for COVID-19

**DOI:** 10.1093/ofid/ofac492.953

**Published:** 2022-12-15

**Authors:** Luis Del Carpio-Orantes, Sergio García-Mendez, Jesús Salvador Sánchez-Díaz, Andrés Aguilar-Silva, Omar García-Hernández, Raúl Enrique Salazar-Lizárraga, América Alejandrina González-Arce, Manuel Martínez-Rojas, Oscar Rodrigo Jiménez-Flores, Luis Roberto Villalobos-López, Héctor Nahín Hernández-Gómez, Rubén Domínguez-Cámara

**Affiliations:** Instituto Mexicano del Seguro Social, Veracruz, Veracruz-Llave, Mexico; Hospital Regional de Alta Especialidad de Oaxaca, Veracruz, Veracruz-Llave, Mexico; Instituto Mexicano del Seguro Social, Veracruz, Veracruz-Llave, Mexico; Instituto Mexicano del Seguro Social, Veracruz, Veracruz-Llave, Mexico; Instituto Mexicano del Seguro Social, Veracruz, Veracruz-Llave, Mexico; Instituto Mexicano del Seguro Social, Veracruz, Veracruz-Llave, Mexico; Instituto Mexicano del Seguro Social, Veracruz, Veracruz-Llave, Mexico; Instituto Mexicano del Seguro Social, Veracruz, Veracruz-Llave, Mexico; Instituto Mexicano del Seguro Social, Veracruz, Veracruz-Llave, Mexico; Instituto Mexicano del Seguro Social, Veracruz, Veracruz-Llave, Mexico; Instituto Mexicano del Seguro Social, Veracruz, Veracruz-Llave, Mexico; Instituto Mexicano del Seguro Social, Veracruz, Veracruz-Llave, Mexico

## Abstract

**Background:**

Baricitinib is a treatment authorized by the FDA for the treatment of moderate to severe COVID-19, despite this there are few approved drugs; polymerized type I collagen (PTIC) is a drug that has been used in Mexico with great potential for treating moderate to severe cases of COVID-19.

**Methods:**

Comparative, descriptive and retrospective analysis of two populations of adult patients affected by COVID-19 confirmed by antigen test or RT-PCR as well as CO-RADS 6 CT, who consented to be treated between 2020 and 2021, a population using oral baricitinib at a dose of 4mg/day/14 days and another using polymerized type I collagen intramuscularly at a dose of 1.5ml every 12 hours for 3 days, followed by 1.5ml every 24 hours for 4 days; The most affected age and gender, comorbidities and laboratory abnormalities are analyzed, as well as improvement in inflammatory and oxygenation indices measured by pulse oximetry and SAFI (SpO2/FiO2), finally the outcome of the patients and the presence of adverse events.

**Results:**

80 patients for each group, the most affected gender was male; the average age in the PTIC group was 51 years and in the baricitinib group it was 56 years; the main comorbidities were obesity, diabetes and hypertension in both groups; the decrease in acute phase reactants such as CRP, D-dimer and ferritin was greater in the PTIC group compared to the baricitinib group, the latter drug requiring a regimen of more days to achieve the objectives of the first drug (PTIC 7 days and baricitinib 14 days); Similarly, in oxygenation measured, the PTIC group reached goals in less time compared to the baricitinib group, which required twice as many days of treatment to achieve adequate oxygenation; Regarding the outcomes, there was a higher mortality in the baricitinib group compared to the PTIC group (6.25% vs 3.75%). Regarding adverse events reported for the PTIC group, they were minor and related to the intramuscular administration of the drug in 7 patients, while in the baricitinib group, 5 patients were reported with added bacterial pneumonia.

**Figure:**
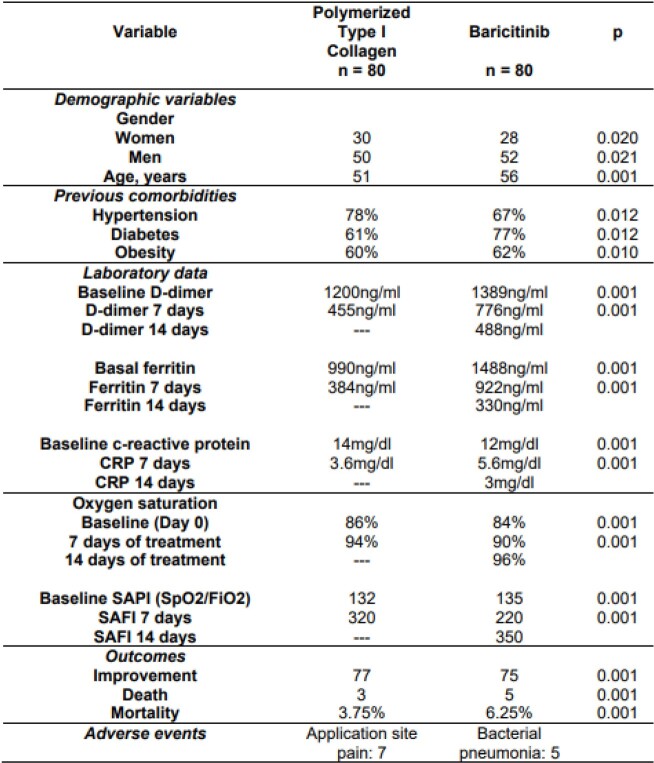

**Conclusion:**

Polymerized type I collagen has anti-inflammatory and immunomodulatory potential similar to baricitinib in cases of moderate to severe COVID-19, even reaching treatment goals in less time both in inflammatory indices and in oxygenation indices.

**Disclosures:**

**All Authors**: No reported disclosures.

